# Diagnostic features and management options for duodenal neuroendocrine neoplasms: a retrospective, multi-centre study

**DOI:** 10.1038/s41598-022-19738-9

**Published:** 2022-09-21

**Authors:** Dalvinder Mandair, Lukasz Kamieniarz, Michail Pizanias, Martin O. Weickert, Akshay Narayan, Luke Furtado O’Mahony, Martyn Caplin, John Ramage, Andreas Prachalias, Rajaventhan Srirajaskanthan, Christos Toumpanakis

**Affiliations:** 1 Neuroendocrine Tumour Unit, ENETS Centre of Excellence, Royal Free Hospital, London, NW3 2QG UK; 2 University College Medical School, London, UK; 3 Neuroendocrine Tumour Unit, ENETS Centre of Excellence, Institute of Liver Studies, Kings College Hospital, London, UK; 4 The ARDEN NET Centre, ENETS CoE, University Hospitals Coventry and Warwickshire (UHCW) NHS Trust, Coventry, CV2 2DX UK; 5 Centre of Applied Biological & Exercise Sciences (ABES), Faculty of Health andLife Sciences, Coventry University, Coventry, CV1 5FB UK; 6 Department of Hepatobiliary Surgery, Institute of Liver Studies, Kings College Hospital, London, UK

**Keywords:** Cancer, Endocrinology, Gastroenterology, Oncology, Risk factors, Signs and symptoms

## Abstract

Duodenal neuroendocrine neoplasms (dNENs) are rare neoplasms but their incidence is on the rise. They are classified into 5 sub-types but there remains much heterogeneity in behaviour in particular of non-functioning dNENs. To retrospectively analyse outcomes for all types of dNENs, and highlight prognostic factors associated with worse outcome. 102 (57 m/45f.) patients were identified with mean age at diagnosis 62 (range 32–87) years. The majority were non-functioning tumours 87/102 and median size was 10 mm (range 0.9–130 mm). 83 patients had Stage I or II disease, of which 17 underwent endoscopic resection with R1 rate of 45% and complication rate 12%. 36 patients were kept under endoscopic surveillance. There were 11 deaths of which 4 were disease related. Age and Ki67 > 20% were associated with worse OS in all dNENs. In non-functioning dNENs Ki67 > 3% was a predictor of lymph nodes metastases with OR 18.2 (2.54–13) (*p* < 0.005) in univariate analyses and liver metastases with OR 6.79 (1.56–29.5) (*p* < 0.05) in the multivariate analysis. Lesions 11–20 mm in size had OR 11.1 (1.16–106) compared to lesions < 11 mm for the prediction of lymph node metastases in the multivariate analysis (*p* < 0.05). ROC analysis of size of non-functioning dNENs to predict LN metastases found < 15 mm had an AUROC of 0.9 (0.81–0.99) with a sensitivity of 85% and specificity of 88%. dNENs are increasing in incidence, however low grade and smaller lesions have an indolent course and the role of endoscopic resection and active surveillance needs to be reviewed.

## Introduction

Duodenal neuroendocrine neoplasms (dNENs) are rare neoplasms with a reported incidence of 0.17 per 100,000^1^. They represent at least 2.7% of all NENs^2^. This figure is on the rise along with the incidence of all NENs which has increased from 1.09 per 100,000 in 1973 population to 6.98 in 2012 according to US based National surveillance, epidemiology and end results database (SEER). The incidence of localised disease has increased from 0.21 per 100,000 to 3.15 per 100,000 in 2012^3^.This rise in NENs detected at an early stage is likely due to the increase in endoscopy investigations being performed and more generous use of scans such as computed tomography in asymptomatic patients^4^.

dNENs are classified into 5 clinical sub-types: non-functioning, functioning consisting of gastrinomas and somatostatinomas, carcinoids (serotonin-secreting), duodenal paragangliomas and poorly differentiated neuroendocrine carcinomas (NECs)^5,6^. Epidemiological studies from the previous decade reported the syndromic subtypes of dNENs (specifically the gastrinomas and the carcinoid tumours) as more prevalent than the non-functioning dNENs^5,7^. In the last 20 years an increasing proportion of non-functioning dNENs are found incidentally at endoscopy and are now the most prevalent sub-type^8^. The average size of the dNENs at diagnosis has been reported to be between 1.2 cm and 1.85 cm in previous studies^5,9^. The incidence of lymph nodes has been reported as high as 40–60%^1^, with liver metastases seen in 10–15%^1,5^. The tumour histology affects the survival: patients with neuroendocrine carcinomas (NECs) have a median survival of just over a year^10^. Other previously identified significant factors in the prognosis of these neoplasms include depth of invasion, size and mitotic activity. Tumours below < 1 cm are rarely associated with distant metastases and local lymph node involvement is seen in less than 5% of patients^11^. Invasion of muscularis propria and high mitotic rate are associated with higher risk for metastatic disease^1^.

The current ENETS dNENs management guidelines recommend endoscopic removal of all non-functioning dNENs < 1 cm^1^. The incidence of complications post endoscopic resection has recently been reported as 8.6% (24/279) in a systematic review of 21 retrospective studies^12^.The proportion of resections with tumour present at the margin (R1) has been reported as high as 40%^13^.

With a high complication rate at endoscopic resection, but also the indolent behaviour seen in many smaller lesions, there is a need for further evaluation of the outcomes of non-functioning dNENs.

The aim of this study was to retrospectively analyse the behaviour and outcomes for all types of dNENs, with an attempt to highlight prognostic factors associated with worse outcome.

## Materials and methods

### Study population

This was multi-centre study retrospective study involving 3 ENETs Centres of Excellence, University Hospitals Coventry and Warwickshire (UHCW audit proposal number 139/2017), Kings College Hospital and the Royal Free Hospital. Patients presented between to these centres between January 2005 and December 2017. 102 patients were identified by searches of NET patient database in each centre. All patients had histological confirmation from either biopsy, endoscopic or surgical resection or from liver metastases. Ampullary NENs were not included in this analysis as their behaviour is more closely associated with that of pancreatic NENs. Patients with synchronous tumours in other sites were also excluded.

### Ethics

All patients included in the study were followed up until July 2018. This was a retrospective study observational study, there were no experiments conducted on the patients and all patients provided informed consent. This study was registered with the Quality Governance and clinical audit committee of the Royal Free Hospital NHS Trust and was conducted in accordance with the Declaration of Helsinki.

### Data analyses

The clinico-pathological records were analysed and data collected using a pre-developed clinical record form. Size, grade, and location of each the duodenal lesion was recorded. Their clinical subtype was also recorded. Where there were multiple lesions, the largest was included in the survival analyses. Histological grade was assessed and according to the WHO 2010 guidelines 2010 for digestive tumours^14^. The presence of lymph node, hepatic and skeletal metastases was assessed, at the time of diagnosis and follow-up, through cross-sectional and molecular imaging (Gallium 68 DOTATATE PET scan or Octreoscan + /− FDG PET). Tumours were staged according to the ENETs TNM classification for gastro-duodenal neuroendocrine neoplasms^15^. All treatments were recorded. Overall survival was defined as time of diagnosis till most recent follow-up July2018. There were few deaths recorded in the follow-up period, therefore in the survival analysis all dNENs were included (high grade and functioning). These groups were excluded from a sub-group analysis of non-functioning well differentiated dNENs where risk factors for lymph node and liver metastases were investigated.

### Statistics

Statistical analyses was performed using Graph pad Prism version 8 and Stata version 16. Categorical variables were presented as frequencies and percentages and continuous variables as means with range. Survival was estimated using Kaplan–Meier methods and cox hazards ratios were calculated to compare differences in survival in patient groups. Logistic regression analysis was performed to determine the risk factors for the presence of lymph node or liver metastases.

## Results

### Patients

There were one and hundred and two patients with complete medical records identified from 3 ENETs centres of excellence. Tumour characteristics are summarised in Table 1. There were a similar number of male and female patients with mean age at diagnosis 62 (range 32–87) years. The majority of tumours were found in D1 (75%) and 87 were non-functioning dNENs (85%). Most patients were found at an earlier stage, with 46 patients at Stage I and 27 at Stage II. The mean size of tumour was 16 mm, consistent with previously reported data, with median 10 mm (range 1–130 mm). All patients that demonstrated FDG-PET avidity in the primary or metastases had a Ki67 of more than 15%.

**Table 1:** Demographics and clinical characteristics of 102 dNENs. PPPD –pylorus preserving pancreatoduodenectomy. SSTA – somatostatin analogues. PRRT – peptide radiolabelled receptor targeted therapy.

Number of patients	102 57 m/45f.
Mean age at diagnosis	62 (32–87)
Location D1	63 (62%)
D2	19 (19%)
D3	3 (3%)
D4	2 (2%)
Unknown	15 (15%)
Grade 1 Ki 67 < 3%	68 (67%)
Grade 2 Ki 67 (3–20%)	21 (21%)
Grade 3 Ki 67 > 20%	3 (3%)
Unknown	10 (10%)
Size	Mean 16 mm (range 0.9–130 mm) Median 10 mm
< 10 mm	43 (42%)
11-20 mm	39 (38%)
> 20 mm	18 (18%)
Unknown	2 (2%)
Subtype—Non-functioning	87 (85%)
Gastrinoma	9 (9%)
Carcinoid	3 (3%)
Paraganglioma	1 (1%)
Poorly differentiated NEC	2
Stage I	48 (47%)
Stage II	21 (21%)
Stage III	8 (8%)
Stage IV	25 (25%)
Non-medical treatment	83 patients
Active surveillance	36 (43%)
Endoscopic resection	17 (20%)
Surgery	30(36%) PPPD 4
	Whipples 8
	Segmental resection 9
	Partial duodenectomy 9
	Synchronous liver mets resection 8 pts
Medical treatment	25 patients
SSTA’s	23 (92%)
Chemotherapy	7 (28%)
PRRT	5 (20%)
Functional imaging avidity	
Gallium PET scan	31/55(56%)
FDG avidity	8/39 (21%)

### Treatments

Patients with Stage I or Stage II disease that was technically resectable were managed with active surveillance in 36 cases. Endoscopic resection (ER) was performed in 17 cases with an incomplete (R1) resection rate seen 45%. Two cases proceeded to surgery (12%) after ER, due to development of perforation. Surgery was performed in a total of 30 cases. Medical therapy was used for patients who presented with Stage IV disease or those that later developed recurrence after previous surgery. The majority received first line treatment with somatostatin analogues (23 out of 25); the exception being those with poorly differentiated NEC, who received chemotherapy. Patients that progressed on somatostatin analogues were given either Peptide Receptor Radionuclide Therapy (PRRT) or chemotherapy and this decision was based on tumour avidity in somatostatin receptor imaging (either Gallium 68 DOTATATE PET scan or Octreoscan).

In the 8 patients that had an R1 resection, further intervention was not performed and patients were kept on active endoscopic and radiological surveillance. This continued until the end of the follow-up period, July 2018. This gave a median follow-up of 31 months (range 26–43).

Treatment options for all patients are summarized in Table 1. Some patients received more than one treatment. There were 5 patients that required medical therapy for recurrence after previous surgery.

### Overall survival

Patients were followed up for median of 49 months (range 61-66) with a mean of 55.9 months (SE + /− 4.27).

At the end of the follow-up period there had been 11 deaths with disease related mortality seen in only 4 patients (4%) and 2 of these patients were poorly differentiated NECs. All dNENs were included in the survival analysis.

Age was a significant predictor of survival in the multivariate cox hazards analysis with a HR 2.1.2 (*p* < 0.05).

Functioning dNENs appeared to fare worse than non-functioning with HR 3.13 (0.85–11.1) (*p* = 0.05) but this was not significant in the multivariate analysis. The summary of cox hazards univariate and multivariate is shown in Table 2.

**Table 2:** Cox-hazards ratio for univariate and multi-variate analysis for OS for dNENs. *Co-linear with Ki67 so calculated in separate multivariate model. See supplementary data.

Characteristic	Univariate HR	Univariate *p*	Multivariate HR	Multivariate *p*
Age	2.62 [1.51–4.54]	*p* = 0.0006	1.96 [0.97–3.96]	*p* = 0.061
Gender				
Female	1			
Male	1.62 [0.49–5.38]	*p* = 0.434		
Size				
< = 10 mm	1			
11-20 mm	1.34 [0.37–4.80]	*p* = 0.654		
> 20 mm	1.09 [0.21–5.54]	*p* = 0.916		
Location				
D1	1			
D2	1.23 [0.31–4.93]	*p* = 0.772		
Ki67				
G1-2	1		1	
G3	12.5 [2.38–65.2]	*p* = 0.003	5.10 [0.79–32.8]	*p* = 0.086
FDG				
Negative	1			
Positive	1.44 [0.15–14.2]	*p* = 0.753		
68 Ga				
Negative	1			
Positive	0.61 [0.13–2.80]	*p* = 0.528		
Functional				
Non-functional	1		1	
Functional	3.32 [0.98–11.2]	*p* = 0.053	2.19 [0.52–9.14]	*p* = 0.284
Liver mets*				
Absent	1		1	
Present	2.87 [0.92–9.00]	*p* = 0.071	2.16 [0.67–6.97]	*p* = 0.196
LN mets				
Absent	1			
Present	1.99 [0.63–6.29]	*p* = 0.239		

**Figure 1:**
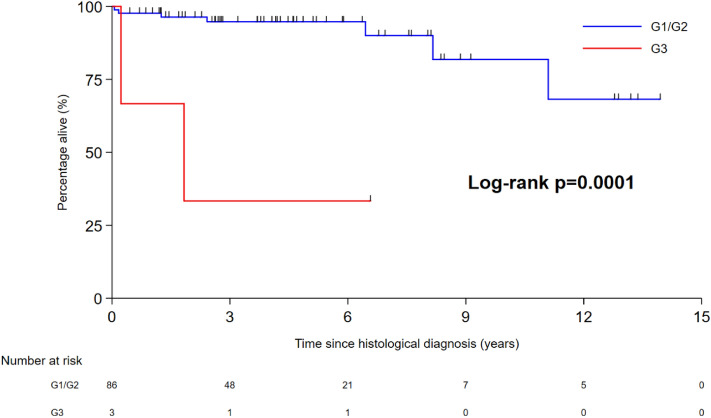
Kaplan–Meier survival curve for dNENs using a Ki67 cut-off of 20% (G3).

Ki67 was a significant predictor of overall survival in the univariate analysis. A Ki67 of 20% was used as a cut-off in the survival analysis. The Kaplan–Meier curve is demonstrated in Fig. 1. The median survival with patients with a Ki67 of > 20% was 1.83 years, whilst that of Ki67 < 20% was not reached (*p* = 0.0001).

Size was not a significant predictor of survival in this analysis because there were too few deaths in the follow-up period. However when adjusted for disease related mortality, the 5 year survival lesion was 100% for lesions less than 1 cm in size.

### Risk factors for metastases

There were 31 patients that were identified to have lymph node metastases. Patients were carefully staged with cross sectional imaging, EUS and Gallium PET. All these patients with evidence of lymph node involvement went on to have surgery except one that opted for medical treatment alone.When partial duodenal resection was undertaken (not the whole circumference) lymph node sampling/clearance would involve retro pancreatic and aorto-caval nodes.

When segmental resection was performed this would extend to involve the DJ flexure and in addition to the 2 groups of lymph nodes above, would also incorporated the short mesentery chain to the left of the superior mesenteric artery.

To identify the risk factors for lymph node and liver metastases, sub-group analysis was performed on non-functioning dNENs as this represented a more homogenous group. The univariate and multivariate logistic regression analysis is summarised in Tables 3 and 4. Ki67 was a predictor of lymph node metastases in the univariate analysis with Ki67 > 3% associated with an odds ratio of 18.2 (2.54–13) with *p* < 0.005. Ki67 was a predictor of liver metastases with an odds ratio of 6.79 (1.56–29.5) with *p* < 0.05 in the multivariate analysis. Size was a key predictor of both lymph node and liver metastases. Lesions that were between 11 and 20 mm in size in size had an odds ratio of 11.1 (1.16–106) compared to lesions < 11 mm for the presence of lymph node metastases in the multivariate analysis (*p* < 0.05). When lesions > 20 mm were compare to lesions < 11 mm, the odds ratio was 16.7(2.08–134) (1.4–33.86) with *p* < 0.01) for prediction of liver metastases in the multi-variate. The only non-functioning dNEN that had lymph node metastases had a Ki67 of 10%.

**Table 3:** Summary of univariate and multi-variate cause logistic regression for all non-functioning dNENs to predict LN metastases.

Characteristic	Univariate OR	Univariate *p*	Multivariate OR	Multivariate *p*
**Age**				
In tens	1.03 [0.71–1.50]	*p* = 0.876		
**Gender**				
Female	1		1	
Male	0.33 [0.12–0.87]	*p* = 0.025	0.05 [0.005–0.52]	***p* = 0.012**
**Size**				
< = 10 mm	1		1	
11-20 mm	7.35 [1.40–38.6]	*p* = 0.018	11.1 [1.16–106]	***p* = 0.037**
> 20 mm	91 [13.7–605]	*p* < 0.00001	457 [17.4–11986]	***p* = 0.0002**
Location*				
D1	1		1	
D2	4.76 [1.30–17.5]	*p* = 0.019	8.58 [1.26–58.6]	***p* = 0.028**
**Grade**				
G1	1		1	
G2-3	19.7 [5.38–72.1]	*P* < 0.00001	18.2 [2.54–131]	***p* = 0.004**
FDG**				
Negative	1		1	
Positive	9.2 [1.30–64.9]	*p* = 0.026	9.23 [1.28–66.8]	***p* = 0.028**
**68 Ga**				
Negative	1			
Positive	2.71 [0.68–10.8]	*p* = 0.158		

*Location was multi co-linear with FDG and size, so was analysed in a separate multivariate model—please see supplementary tables. **FDG was multi co-linear with grade, location and size, so was analysed in a separate multivariate model—please see supplementary tables.

**Table 4:** Summary of univariate and multi-variate analysis for all non-functioning dNENS to predict liver metastases.

Characteristic	Univariate OR	Univariate *p*	Multivariate OR	Multivariate *p*
**Age**				
In tens	1.05 [0.70–1.57]	*p* = 0.829		
**Gender**				
Female	1		1	
Male	0.35 [0.12–1.01]	*p* = 0.052	0.24 [0.05–1.10]	*p* = 0.067
**Size**				
< = 10 mm	1		1	
11-20 mm	5.86 [1.09–31.6]	*p* = 0.040	5.16 [0.76–35.2]	*p* = 0.093
> 20 mm	26.4 [4.68–149]	*p* = 0.00003	16.7 [2.08–134]	***p* = 0.008**
Location*				
D1	1		1	
D2	8.67 [2.02–36.9]	*p* = 0.004	23.0 [2.26–234]	***p* = 0.008**
**Grade**				
G1	1		1	
G2-3	16.2 [4.50–58.3]	*p* = 0.00002	6.79 [1.56–29.5]	***p* = 0.011**
FDG**				
Negative	1		1	
Positive	6.00 [0.88–40.9]	*p* = 0.067	6.09 [0.89–41.9]	*p* = 0.066
**68 Ga**				
Negative	1			
Positive	1.40 [0.32–6.07]	*p* = 0.655		

*Location was multi-collinear with FDG and size, so was analysed in a separate multivariate model—please see supplementary tables. **FDG was multi-collinear with grade, location and size, so was analysed in a separate multivariate model—please see supplementary tables.

To identify the optimal cut-off for the prediction of lymph node metastases ROC (receiver operating characteristic) analysis was performed for dNENs that were of low grade and non-functioning. Lesions less than 15 mm had an AUROC of 0.

**Figure 2:**
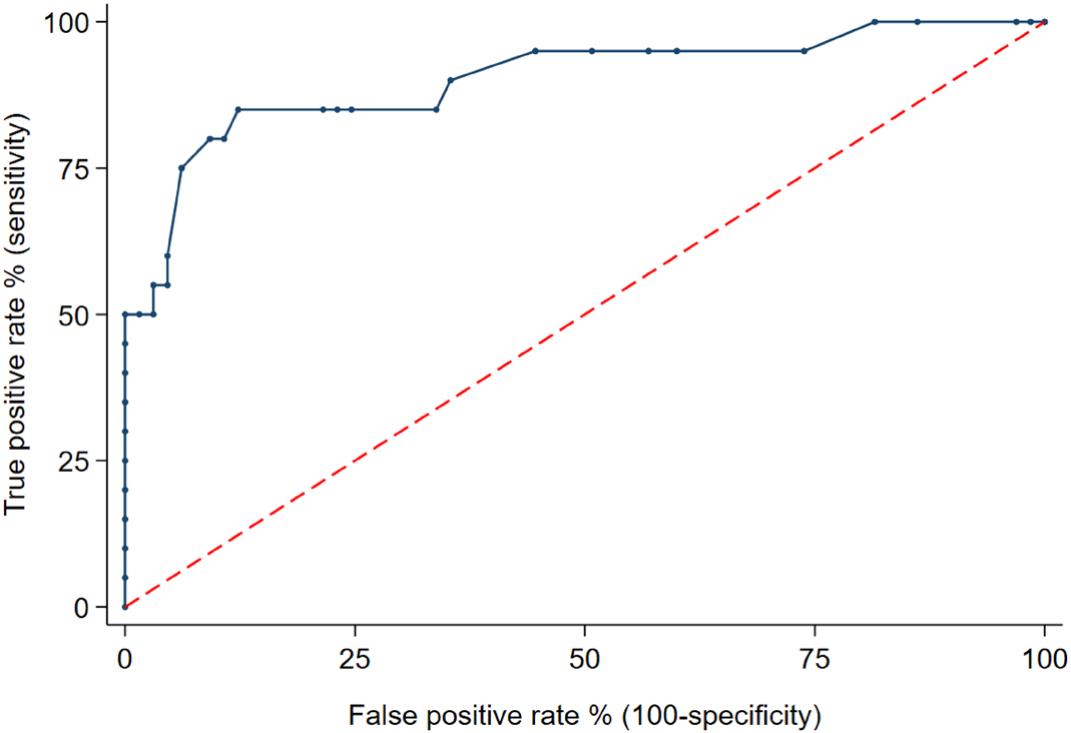
AUROC analysis for size of dNENs as a predictor of LN metastases in non-functioning d NENs.

9 (0.81–0.99) with a sensitivity of 85% and specificity of 88% for the prediction of lymph node metastases (Fig. 2).

## Discussion

Overall dNENs have an excellent prognosis. Our finding of only 4 deaths with disease related mortality is similar to previous retrospective studies that have demonstrated an overall 5-year survival for all dNENs has been reported at 85% with median overall survival of 187 months^8,16^. The 5 year survival for lesions less than 1 cm in size was 100% in our study.

This study has further demonstrated the changing spectrum of dNENs. There were 87/102 (85%) non-functioning dNENs, compared to 106/166 (63.6%) in an earlier retrospective study by Vanoli et al.^17^. A more recent retrospective series by Massironi et al. found 78/108 (72%) were non-functioning^8^.

The largest published data on dNENs looked at 1,258 identified from SEER found an increasing proportion of patients found at an earlier stage (Stage I-II) increased from 65% 1983–2005, to 79% in 2006–2010^18^. This was mirrored in our study as 71% of patients were found between Stage I–II.

Current ENETS guidelines recommend endoscopic removal for all lesions that are < 10 mm in size and surgery for all lesions that are > 20 mm in size, T2 or if there is incomplete resection. For lesions between 10 and 20 mm without nodal spread there was no overall consensus as either endoscopy or surgery could be considered in these patients^15^. The guidelines do emphasise the need for careful staging with both EUS and cross-sectional imaging prior to any attempts at endoscopic resection.

Endoscopic resection was performed in 17 patients with 8 patients having an incomplete R1 resection, and two patients suffering a perforation and requiring emergency surgery. There were no clear predictors with regard to size or location that could predict an incomplete resection or a perforation. A retrospective study of 38 dNENs measuring < 10 mm by Kim et al. found 17/38 patients (41%) had a clear histopathological resection margin. The four cases that underwent endoscopic sub-mucosal resection (ESD) all had a clear resection margin^13^. The duodenum is thin walled and has a narrow lumen making endoscopic resection more challenging and associated with significant risk of perforation and bleeding^19^. ESD is associated with far greater risk than in the lower GI tact and perforation has been reported in up to 30% of cases in the duodenum^12^.

In our case series, 36 patients that could have been considered for ER or surgical resection were placed on active surveillance alone. They underwent annual endoscopic surveillance and cross sectional imaging. There was no progression seen in these patients and 5 year survival was 100%. The prognosis for non-functioning dNENs without local or distant metastases is very good. The relatively high complication rate and incomplete resection with ER, and the worse quality of life reported after surgical resection^11^, it may be lesions that show such indolent behaviour are best kept on surveillance alone.

To help identify which lesions could be considered for observation alone we looked at factors that could be predictive of lymph node and liver metastases in non-functioning dNENs. In the logistic regression analysis, Ki67 > 3% and size > 2 cm were the strongest predictors of lymph nodes metastases. In ROC analysis of G1 non-functioning dNENs a cut-off of < 15 mm in size had an AUROC of 0.96 with a sensitivity of 92% and specificity of 89%. A study by Park et al. that analysed 44 dNENs all removed at endoscopy in 38 patients found size > 10 mm, non-bulbar location, invasion beyond sub-mucosa (found at EUS) and lymphovascular invasion were predictors of LN metastases^20^. Vanoli et al. found similar findings with regard grade, submucosal penetration and grade. In their ROC analysis they found a cut-off of 0.9 cm had an AUROC of 0.79, with sensitivity of 84% and specificity of 73%, however they included G2 dNENs in their analysis^17^.This study has a number of limitations due to its retrospective nature looking at outcome and treatments across 3 different centres with varying approaches to in particular endoscopic management. As dNENs are often slow growing, there were very few deaths therefore the survival data has to be interpreted with caution. There was also data missing from findings at endoscopic ultrasound.

It was difficult to determine progression as a surrogate end point as currently there is no definition of what would be deemed endoscopic progression for dNENs that were being kept under surveillance without any radiologically measurable disease.

Nevertheless, the findings of this study concur with recent publications documenting the rise in incidence of non-functioning dNENs, their good prognosis and the risk factors associated with more aggressive disease. There is a cohort of patients that could be considered for active surveillance alone but this needs further evaluation in large, long-term multi-centre prospective settings.

**Figure 3:**
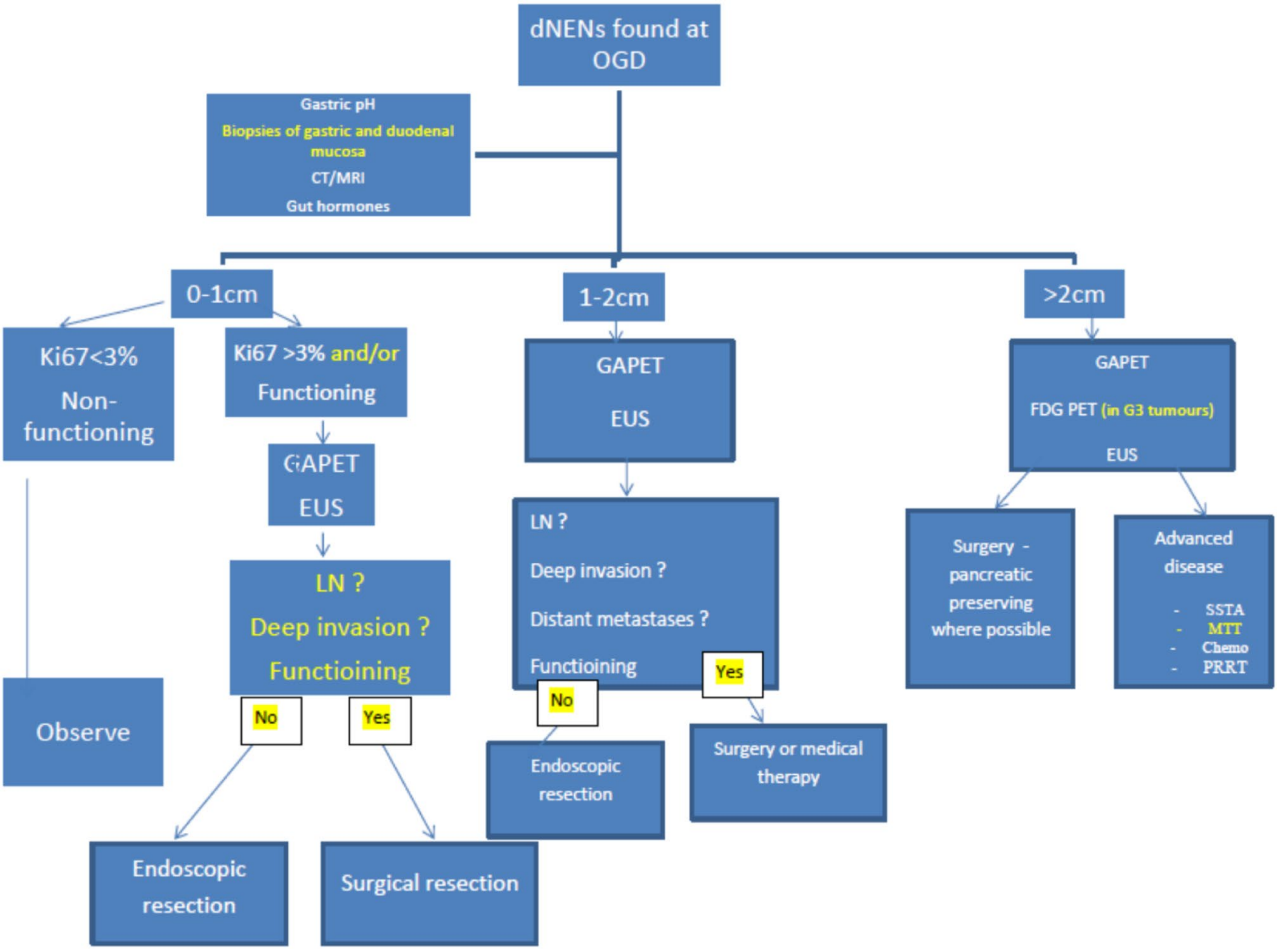
Proposed management algorithm for duodenal NENs. dNENs: duodenal neuroendocrine neoplasms, *CT* Computer tomography, *MRI*: Magnetic resonance imaging *LN* lymph nodes, *EUS* Endoscopic ultrasound, *GAPET* Gallium68 DOTATATE Positron emission tomography FDG PET Flurodeoxyglucose positron emission tomography. *SSTA* Somatostatin analogue *MTT* molecular targeted treatment PRRT Peptide receptor radio-targeted therapy, *G3* Grade 3 Ki 67 > 20%.

In conclusion, although dNENs appear to be increasing in incidence, this seems to be primarily made up of non-functioning dNENs which are associated with a very good prognosis. The role of endoscopic resection for dNEN that are associated with good outcome, (low grade, non-functioning and less than 11 mm in size) needs to be reviewed. There may be a role for new endoscopic techniques such as full thickness resection which may have better R0 resection rates and less risk of complications^21^ From our experience across three ENETS centre of excellence we have developed an algorithm for the management of non-functioning dNENs which could prevent unnecessary intervention associated with significant morbidity but also ensure adequate surveillance still takes place where indicated (Fig. 3).

## Supplementary Information

Supplementary Information.
