# Supplementary material for: Diagnostic features and management options for duodenal neuroendocrine neoplasms: a retrospective, multi-centre study
Source: Sci Rep. 2022 Sep 21;12:15762. doi: 10.1038/s41598-022-19738-9 (PMC9492724; doi:10.1038/s41598-022-19738-9)
Supplement: Supplementary file 1 — Supplementary Information. [file 41598_2022_19738_MOESM1_ESM.docx]

|  | Multivariate HR | Multivariate p |
| --- | --- | --- |
| **Age** | **2.44 [1.37-4.33]** | **p=0.002** |
| Functional  Non-functional  Functional | 1  2.49 [0.71-8.74] | p=0.156 |
| Liver mets*  Absent  Present | 1  2.16 [0.67-6.97] | p=0.196 |

Table S1 – supplementary data with separate multivariate analysis for table 2

| Characteristic | Multivariate OR | Multivariate p |
| --- | --- | --- |
| Gender  Female  Male | 1  0.53 [0.10-2.89] | p=0.463 |
| FDG**  Negative  Positive | 1  **9.23 [1.28-66.8]** | **p=0.028** |

| Characteristic | Multivariate OR | Multivariate p |
| --- | --- | --- |
| Gender  Female  Male | 1  0.49 [0.09-2.72] | p=0.415 |
| **Location***  D1  **D2** | 1  **8.58 [1.26-58.6]** | **p=0.028** |
| **Grade**  G1  **G2-3** | 1  **27.8 [4.31-180]** | **p=0.0005** |

Table S2 – supplementary data with separate multivariate analysis for table 3

| Characteristic | Multivariate OR | Multivariate p |
| --- | --- | --- |
| Gender  Female  Male | 1  0.49 [0.09-2.72] | p=0.415 |
| **Location***  D1  **D2** | 1  **8.58 [1.26-58.6]** | **p=0.028** |
| **Grade**  G1  **G2-3** | 1  **27.8 [4.31-180]** | **p=0.0005** |
| Characteristic | Multivariate OR | Multivariate p |
| Gender  Female  Male | 1  0.77 [0.11-5.26] | p=0.788 |
| **Location***  D1  **D2** | 1  **23.0 [2.26-234]** | **p=0.008** |
| **Grade**  G1  **G2-3** | 1  **23.8 [2.34-241]** | **p=0.007** |

Table S3 – Supplementary data with separate multivariate analysis for table 4
